# Effect of the emotional valence of autobiographical memory and parental bonding on depressive symptoms in a community sample

**DOI:** 10.1038/s41598-023-33916-3

**Published:** 2023-04-25

**Authors:** Dolores Fernández-Pérez, Laura Ros, María V. Jimeno, José Miguel Latorre

**Affiliations:** 1 Department of Psychology, Faculty of Medicine, University of Castilla-La Mancha, Albacete, Spain; 2 Neurological Disabilities Research Institute, Albacete, Spain

**Keywords:** Psychology, Human behaviour

## Abstract

Retrospective perceptions of parental bonding may be a protective factor for emotional health. These perceptions are grounded in autobiographical memory, which plays a key role in the onset and maintenance of depressive symptomatology. The aim of the present study was to explore whether the emotional valence of autobiographical memories (positive and negative) and the dimensions of parental bonding (care and protection) have an impact on depressive symptomatology, examining the role of depressive rumination and possible age-related differences. A total of 139 young adults (18–28 years) and 124 older adults (65–88 years) completed the Parental Bonding Instrument, the Beck Depression Inventory (BDI-II), the Autobiographical Memory Test and the Short Depressive Rumination Scale. Our results show that positive autobiographical memories serve as a protective factor for depressive symptoms in both young and older adults. Additionally, in young adults, high paternal care and protection scores are associated with increased negative autobiographical memories, although this association has no effect on depressive symptomatology. In older adults, high maternal protection scores are directly related to greater depressive symptomatology. Depressive rumination significantly increases depressive symptoms in both young and older adults, with an increase in negative autobiographical memories in young adults, and a decrease in such memories in older adults. Our findings progress the understanding of the relationships between parental bonding and autobiographical memory with respect to emotional disorders, which, in turn, will help in the design of effective prevention measures.

## Introduction

Depression is an emotional disorder^1^ that affects almost 300 million people, causing an enormous global disease burden ^2^. Specifically, depressive symptomatology refers to the group of symptoms that categorise depression, including aspects such as low mood or loss of pleasure in everyday activities^3^. Risk factors that may contribute to the onset and maintenance of depressive symptomatology include autobiographical memories as well as dysfunctional parental bonding^4^.

### Parenting styles and emotional disorders

Among the risk and protective factors for depressive symptomatology, parental bonding holds a key place. This concept refers to a person’s interpretation of their relationship with their parents^5^. According to Bowlby's Attachment Theory^6^, from the early years of their lives, individuals have an innate tendency to establish intimate and stable bonds (of physical and emotional attachment) with people who are significant to them (father/mother). Parenting styles are a fundamental component within this parent–child interaction, with an extensive and enduring impact on the behavioural, social, and emotional development of the child^7^. Previous research has underlined the relationship between poor parental bonds and the development of psychopathological disorders, such as depression, during adulthood^8^.

It is worth clarifying here the differences between attachment and parental bonding. Although both concepts are based on the quality of the relationship between parent and child, each understands the connection between the two parties from a different viewpoint. In this sense, attachment considers a person's perceptions of their current close relationships with peers^9^, whereas parental bonding refers to the perceptions of the first relationships a person establishes with their parents^10^.

Parker^11^ outlined two dimensions of parental behaviour in relation to parental bonds: care and protection. Care is defined, at one pole, as affection, emotional containment, empathy and closeness and, at the other, as emotional coldness, indifference and negligence. Meanwhile, protection may be defined by the absence of control, or, in contrast, by intrusion, excessive contact, infantilization and prevention of independent behaviour. Consequently, the care dimension contrasts with indifference and rejection, while the protection dimension hinders an individual’s capacity to achieve independence and autonomy^12^. Both dimensions contribute to the bond that develops between parent and child during the early stages of life, with an optimal bond being one where parental care is high (emotional warmth and acceptance) and overprotection (psychological control and intrusion) is low^13^. Although parental bonds tend to remain relatively stable and strong^14^, some studies report a deterioration of bonding in the early and middle stages of adolescence, as well as an improvement in late adolescence and early adulthood^15^.

Bowlby^16^ related poor or pathological parenting to the dimensions of lack of care, and excessive control or protection^17^, such that parents who are unable to establish warm, loving and close relationships with their children or who fail to provide an environment that fosters their children's independent development, ultimately raising them in an atmosphere of anxiety and insecurity that may lead to the development of psychological disorders^18^. The influence of parenting styles on individuals is typically formed before the age of 16, with little change observed thereafter, therefore, dysfunctional parental attachment might be a significant risk factor for emotional dysregulation in adulthood^4^.

Consequently, many studies have explored the relationship between parental bonding and the development of depressive symptoms during adulthood^19–21^. The studies conducted, taking into account the dimensions proposed by Parker^10^, report a stronger and more consistent association between the care dimension (specifically lack of care) and the emergence of mood disorders, compared to the protection dimension^19^. The findings of previous research suggest that dysfunctional paternal and maternal parenting styles, characterized by low levels of both care and protection (neglectful parenting), are associated with emotional disorders such as depression^22^. Similarly, low maternal care has been found to be a significant risk factor for the development of clinical depression^23^. In addition, low levels of both maternal and paternal care were associated with suicide attempts in persons with depression^24^. Additionally, high levels of dependency (stemming from overprotection) have been associated with interpersonal problems and cognitive depressive symptoms^25^ due to, among other factors, high levels of submissiveness. Additionally, high overprotection (measured using the PBI) has been associated with increased depressive symptoms in adults (albeit mediated by neuroticism)^26^. Other studies in the same line report a direct effect of high levels of maternal overprotection (but not paternal overprotection) on higher levels of depressive symptomatology in young persons^21^.

Therefore, the quality of parenting an individual is exposed to during the early years of life has effects on their health and well-being^27^. Although this quality is assessed by asking individuals about their experiences during childhood and adolescence, and these perceptions may vary over time, studies suggest that such perceptions remain relatively stable from adolescence to adulthood^28^. In this sense, low quality retrospective evaluations of parenting styles during childhood have been associated with higher levels of negative emotional outcomes during adulthood, including symptoms of depression^29^.

### Autobiographical memory and emotional disorders

Parental bonds and parenting styles received early in our lives shape our expectations and beliefs about ourselves, the world around us, and our social relationships^30^. Retrospective perceptions of these attachments are grounded in Autobiographical Memory (AM), which refers to explicit memories of past events (single, recurrent, and extended events) in a person’s life that have a high emotional impact and personal importance^31,32^. The development of AM is the result of a social process (greatly influenced by the memory of the bonds established with parents)^33^, as well as psychological and neurodevelopmental processes. AM has important functions related to the sense of continuity and coherence of the self over time^34^, to planning and directing our behaviour^35^, and to social bonding^36^. In addition, AM is closely related to affect, shaping emotional states in our everyday lives^37^.

Autobiographical memories, in the same way as the life events they represent, are characterized by a wide range of emotions and emotional reactions^38^. Previous studies have suggested that autobiographical memories are, broadly speaking, biased toward positive events^39^, that is, individuals more frequently access the memory of positive events than of negative events. This is commonly known as the positivity bias and is primarily associated with promoting mental well-being^40^. In the case of memories of negative events, these have great value as alarm and danger signals, being of greater assistance than positive memories in orienting and guiding future behaviour and in avoiding situations similar to past experiences^40^. Nonetheless, access to negatively valenced autobiographical memories is one of the three key domains (together with involuntary/intrusive memories of negative experiences and difficulty accessing specific memories) that impact on the onset, maintenance and recovery of depressive symptomatology^41^. In this sense, an excessive generation of negatively valenced autobiographical memories can lead an individual to develop a negative sense of self, reinforcing and perpetuating negative self-schemas that are characteristic of emotional disorders such as depression^42^.

However, it is worth noting that these negative biases are produced through the retrieval of a memory that is congruent with the person's current mood, matching the emotional valence of the memory with that of the affective state.^43^. Thus, we are dealing with a bidirectional relationship between access to negative autobiographical memories and depressive symptomatology. That is, greater access to negatively valenced personal memories favours the development of depressive symptomatology, in the same way that poor psychological well-being characterised by depressive symptoms leads to a selective memory bias through greater access to negative autobiographical memories and their negative interpretation^44^.

Younger and older adults may present differences in the phenomenology of their autobiographical memories. In this regard, several studies have found that older adults rate their memories as more vivid, coherent, easily accessible, and emotionally intense than do younger adults^45^. Furthermore, older adults recall fewer single or specific events compared to younger people, and, in contrast, access a greater number of general events that are repeated or extended over time^46^. Additionally, older individuals tend to see things from a semantic viewpoint^47^, elaborating their memories less and replacing episodic knowledge with semantic or factual knowledge about their own life story and the context in which the event recalled took place^48^.

Additionally, greater self-reported positive affect has been found in older adults' narratives compared to those of younger adults^45^, as well as more positively valenced memories in older compared to younger people^49^. Furthermore, young adults present a greater tendency to remember negative information rather than positive information^50^, while the opposite is true of older adults^51^. In this sense, the frequency with which emotions are experienced varies with age, with older adults reporting fewer negative emotions than younger adults^52^. That is, older adults show a greater preference for the positive over the negative, which is known as the positivity effect^53^ or the reduced negativity effect^54^. This age-related bias has an impact on autobiographical recall since the retrieval of such memories involves an active process of reconstruction, drawing on the combination of sensory information, narratives, language and emotion^55^. It also contributes to improvement of mood through an increase in positive emotions, particularly in older adults^56^.

#### Depressive rumination and autobiographical memory

In terms of depression, rumination is conceptualized as an emotion regulation strategy^57^ that is able to increase or maintain an affective state^58^. It can be regarded as a repetitive and passive focus on the causes and consequences of one's symptoms of distress without engaging in active coping or problem solving to alleviate dysphoric mood^59^. Specifically, depressive rumination is the tendency to repetitively analyse oneself, one's problems, concerns, feelings of distress and depressed mood^60^. In relation to depressive symptoms, the aim of rumination is to avoid negative emotions associated with the content of autobiographical memories^61^. Nonetheless, it has been suggested that rumination contributes to the worsening of mood and, thus, to the onset and maintenance of depressive symptomatology^62^. Consequently, ruminatively recalling a negative or adverse event could have an emotional impact that might trigger the onset of depressive symptomatology^63^.

The model of capture and rumination, functional avoidance and impaired executive control (CaR-FA-X model)^64^ proposes that individuals who ruminate are more likely to retrieve general autobiographical memories due to the cognitive load involved in ruminative thinking^64^. Overgeneral autobiographical memory (OGM) refers to the difficulty in accessing specific autobiographical memories (events that occurred at a particular place and time and that lasted less than a day), which constitutes a vulnerability factor for the appearance of depressive symptoms and episodes^65^. However, the relationship between rumination and the valence of autobiographical memory has been the subject of scant research^66^, although it seems logical to reason that rumination, as it increases depressive symptomatology, tends to be preferentially associated with negatively valenced memories.

Little research has been conducted on age-related differences in the effect of rumination on access to autobiographical memories and its influence on mood. However, studies have reported that young adults present a higher frequency of rumination compared to older adults^67^. Moreover, the basis of these differences lies in goals related to information gathering across the lifespan, i.e., reflection on negative past events is greater in young people since, in this age group, individuals gather information to avoid future mistakes, even as it increases vulnerability to depression^58^.

### Parenting styles, autobiographical memory and emotional disorders

Retrospective perceptions of parent–child relationships based on AM impact on different dimensions of emotional health^68^. Moreover, autobiographical recall of these parental bonds remains stable over time^69^. To the best of our knowledge, no studies have explored the dimensions of parenting styles (care and protection) and autobiographical recall in relation to depressive symptomatology. However, various studies have focused on parental bonds or attachment relationships (derived from parenting styles) and autobiographical memories, reporting that insecure attachment tendencies (characterized by anxiety and avoidance) are associated with a greater number of negative autobiographical memories^70^. In the same line, several studies have shown relationships between secure attachment styles and the recall of events related to feelings of warmth, and between insecure attachment styles and negative memories^71^.

### The present study

Depressive symptomatology has significant consequences for people, affecting their interpersonal relationships and social performance, which, in turn, leads to impaired quality of life. Considering that the scientific evidence suggests that the family, and more particularly, an individual’s parents, have a vast impact on the development of this symptoms, the aim of the present work is to explore whether the emotional valence of autobiographical memories (positive and negative) and the dimensions of parental styles (care and control/protection) have an effect on depressive symptomatology.

The present study seeks to perform this analysis in a sample of young and older adults on order to study possible age-related differences. In addition, we take into account that research on parenting styles and their link to mental health has largely focused on clinical populations^22^. However, we believe it to be of interest to study all the variables that might contribute to vulnerability for the development of emotional disorders in non-clinical population and thus design effective prevention measures.

## Method

### Participants

The study sample comprised 263 participants (179 women and 84 men). The first groups were formed by 139 young adults (67.6% female) aged between 18 and 28 (*M* = 19.57, *SD* = 1.86). Of these, 97.8% were students and 2.2% were employed. Regarding level of education, 95.6% had completed secondary school and 4.4% had a university degree. Our second group consisted of 124 older adults (68.5% women) aged between 65 and 88 years (*M* = 72.26, *SD* = 5.59). As regards their employment status, 77.4% were retired or pensioners, 26.9% were homemakers, 4.8% were employed and 0.8% were self-employed. With regard to educational level, 41.9% had completed secondary education, 27.4% had completed primary education, 25% had a university degree and 5.6% had completed no formal education. No statistically significant gender-related differences were observed between the age groups (*χ*2(1) = 0.026, *p* > 0.050).

The participants in the group of older adults were recruited from sociocultural centres in the city of Albacete, while the participants in the group of young adults were recruited from among students at the University of Castilla-La Mancha enrolled in degree courses in medicine, law and education on the Albacete campus. The inclusion criteria were as follows: (1) no cognitive impairment, in the case of older adults (assessed using Test Your Memory (TYM)^72,73^; (2) absence of clinical symptoms of depression and/or anxiety (measured using PROMIS-Depression and PROMIS-Anxiety)^74^; (3) absence of sensory deficits that might hinder performance on the experimental tasks; (4) having sufficient literacy skills to understand and complete the different tests; and (5) giving signed informed consent.

The study protocol was approved by the Clinical Research Ethics Committee of the Castilla-La Mancha Health Service (protocol number 06/2016). All the participants gave their written informed consent in accordance with the Declaration of Helsinki.

## Materials

### Sociodemographic questionnaire

This was specifically designed for the purpose of the present study, collecting data on age, sex, marital status, level of education and employment status.

### ***Test your memory (TYM)***^73,75^

This cognitive performance test (orientation, sentence copying, semantic information, calculation, verbal fluency, similarities, confrontation, naming and perception), is used in the detection of Alzheimer's disease (AD) and mild cognitive impairment. It consists of 10 tasks (with different scores), with a possible maximum score of 50. The cut-off points established are equal to or less than 36 (dementia). The reliability of the Spanish version is α = 0.86^75^.

### ***Patient-reported outcomes measurement information system -A (PROMIS®-Anxiety 4, short form)***^74^

This questionnaire forms part of the PROMIS-29^76^ and consists of four items to measure anxiety symptoms experienced by an individual in the last seven days. The responses range from 1 = never to 5 = always, where the higher the score, the higher is the anxiety. Total scores range from 4 to 20. The cut-off point was set at 11 points (https://healthmeasures.net). The reliability of this instrument is α = 0.96^74^, with α = 0.76 in the present study.

### ***Patient-reported outcomes measurement information system -D (PROMIS®-Depression 4, short form)***^74^

This questionnaire forms part of PROMIS-29^76^ and consists of four items used to measure the negative affect experienced by an individual in the last seven days. Responses range from 1 = never to 5 = always, where the higher the score, the higher is the negative affect. Total scores range from 4 to 20. The cut-off point was set at 11 points (https://healthmeasures.net). The reliability of this instrument is α = 0.96^74^, with α = 0.77 in the case of this study.

### ***Parental bonding instrument (PBI)***^10^

This instrument assesses offspring’s retrospective perceptions of their parents’ style of affection and attachment to them, focusing on the first 16 years of their lives^9^. It is designed for use with persons aged over 16 years of both sexes and consists of 25 statements divided into two subscales, Care (12 items) and Protection (13 items), which are scored on a Likert-type scale from 0 (“never or almost never”) to 3 (“always or almost always”). In addition, it allows for a separate assessment of the two parents. The total score on the care subscale ranges from 0 to 36 points, while, on the protection subscale, it ranges from 0 to 39 points, such that the higher the score, the greater are the levels of care and protection. It presents good psychometric properties^10^ including its long-term stability^77^. In this study, the reliability for the maternal scale was α = 0.88 (care) and α = 0.84 (protection) and, for the paternal scale, α = 0.89 (care) and α = 0.86 (protection).

### Autobiographical memory test (AMT)

This test follows the procedure established by Williams et al.^78^, using the cue words from the original test designed by Williams and Broadbent^79^ test, that is, three positive keywords (friendship, excitement, smile), three negative keywords (failure, worry, illness) plus two neutral keywords that are used as a test (car, tree). Participants are presented with the different cue words (alternating positive and negative valence; the order was the same for all the participants, with the test starting with a positive word) and instructed to recall a specific memory. Before starting, participants are explained that a specific memory refers to an event, occurrence, or experience that happened to them in a particular place and that lasted a maximum of one day. Participants are given 60 s to access the autobiographical memory, after which, they are asked to describe the memory in writing.

In our study, two independent researchers were categorized the memories generated according to the valence of the memory (positive and negative). The inter-investigator reliability was 93%.

### ***Beck depression inventory II (BDI-II)***^80,81^

This self-report scale comprises 21 Likert-type items and is designed to detect depressive symptoms in adults. It measures the most frequent clinical symptoms, such as anhedonia, sadness, loss of energy and interest, changes in eating and sleeping patterns, loss of concentration and suicidal ideation, among others the respondent has experienced over the last two weeks. The total scores range from 0 to 63 points (the higher the score, the more severe are the depressive symptoms), with scores between 10 and 15 points being established as in the dysphoric range and those above 16 points as in the depressive range^82^. It has a reliability of α = 0.83^81^, and the reliability in the present study was α = 0.83^81^.

### ***Short depressive rumination scale (SDRS)***^83,84^

This scale measures the frequency of rumination in sad or depressed situations in community samples. It consists of 4 items from the Leuven Adaptation of the Rumination on Sadness Scale (LARSS)^85^: (1) “I have difficulty getting myself to stop thinking about how sad I am”, (2) “I get absorbed in thinking about why I am sad and find it difficult to think about other things”, (3) “I repeatedly try to figure out, by doing a lot of thinking, what might be the causes of my sadness”, (4) “I keep thinking about how I feel, to understand myself and my sad feelings better”. These items are rated on a scale from 0 (never) to 10 (very often), according to the frequency with which the person experiences the situations described, with the total score thus ranging from 0 to 40. The scores obtained in this scale are highly correlated with those of widely used scales, such as the Rumination on Sadness Scale (RSS)^86^ and the Ruminative Response Scale (RRS)^87^. In addition, it presents an excellent internal consistency of 0.93^84^. In this study, reliability was α = 0.89.

### Procedure

The participants that met the initial inclusion criteria were assessed in a single session. Before administering the tests, the participants were explained the aims of the study and the procedure to be followed. They were also informed that participation was voluntary and confidential, and that the data generated would only be used for academic and scientific purposes. The instruments were administered individually by a single researcher in a laboratory. The participants began by completing the AMT, which was then followed by the other questionnaires, all of which were completed in approximately 30 min. The researcher was present while the questionnaires were administered so that any participants’’ doubts or concerns could be addressed. None of the participants was excluded.

### Data analysis

Statistical tests were performed using the SPSS 28.0 program. First, t-test and chi-square tests were performed to determine whether there were any significant differences in variables by age group.

A path analysis was then generated separately for young and older groups, using the AMOS 24.0 software to estimate a model of the causal relationships between rumination, PBI subscales (father/mother care and protection), positive and negative autobiographical memories and depressive symptoms. The maximum likelihood method was used to estimate all the parameters of the model. The model fit was assessed by root-mean-square approximation (RMSEA) and the comparative fit index (CFI). As regards RMSEA, values lower or equal to 0.08 represent a reasonable fit and values lower or equal to 0.05 represent a good fit^88^. According to Bentler^89^, models with statistically non-significant *χ*^*2*^ and CFI values greater or equal to 0.09 are indicative of an acceptable fit.

Mediation was examined with simultaneous mediation by multiple variables with the nonparametric bootstrapping sampling procedure^90^. This method uses the data as a surrogate population and takes samples of the sample with replacement. The estimate of each of the samples is the bootstrap distribution, which is a more accurate representation of the population that makes fewer assumptions about the normal distribution. The mediation model tested whether the PBI subscales had indirect effects on depressive symptomatology through positive and negative autobiographical memories. This model was chosen drawing on previous studies analysing the impact of the perception of parental bonding on depressive symptomatology, studying the role of possible moderating variables^21,91,92^. The bootstrapping sampling procedure was performed with software AMOS. Bias-corrected 95% confidence intervals on 2000 bootstrap samples were estimated for all direct and indirect effects.

## Results

### Preliminary analysis

Table 1 shows the descriptive statistics of the variables assessed by age group. The results from the t-tests showed that the young participants presented higher scores in depressive symptoms, rumination, and maternal care scales than the older group. Only 16% of the total number of participants exceeded the cut-off point for mild depressive symptomatology (> 16 points); 21.6% of the young adults and 9.7% of the older ones. Nevertheless, older participants scored higher on the paternal protection scale than younger ones. Finally, regarding the emotional valence of the autobiographical memories, the results suggest that younger individuals remembered more negative memories and less positive memories than their older counterparts. Furthermore, in most cases, the emotional valence of the autobiographical memory was found to correspond to the emotional valence of the word used. This was the case for 68.8% of the words with a positive emotional valence and 70.6% of those with a negative emotional valence. Furthermore, overall, 49.4% of the autobiographical memories had a positive emotional valence while 50.6% had a negative emotional valence. Furthermore, of the total number of participants, 5.7% had only negatively valenced autobiographical memories, while 4.1% presented exclusively positively valenced memories.

**Table 1:** Means and (standard deviations) for the main variables in the study by age group.

Variable	Young group (*n* = 139)	Older group (*n* = 124)	Cohen’s *d*
Depressive symptoms	12.29 (7.85)	8.65 (6.29)	0.51**
Positive memories	2.02 (1.16)	2.48 (0.90)	0.44**
Negative memories	1.63 (0.79)	1.41 (0.80)	0.28*
Depressive rumination	18.40 (9.84)	11.90 (10.32)	0.64**
Parental bonding			
Maternal protection	12.57 (6.74)	14.15 (7.89)	0.22
Paternal protection Mother	10.26 (7.11)	12.45 (7.64)	0.30*
Maternal care	30.16 (5.95)	25.03 (7.65)	0.75**
Paternal care	25.27 (8.68)	23.37 (7.08)	0.24

**p < .001; *p < .05.

### Path analysis

**Figure 1:**
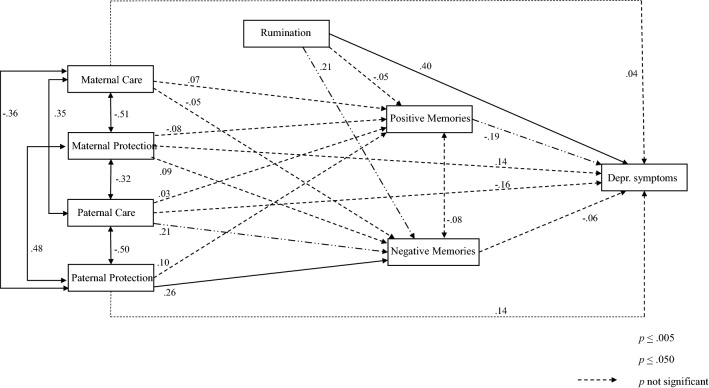
Final structural equation model for young adults.

**Figure 2:**
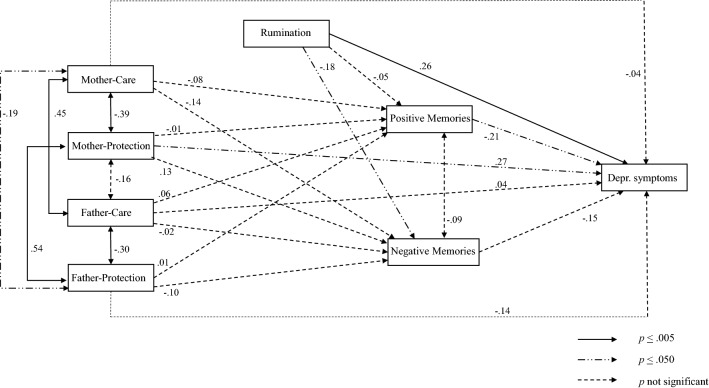
Final structural equation model for olderadults.

Separately for each age group, a path analysis model was used to determine to what extent depressive symptomatology could be explained by parental bonding variables (father/mother care and protection), rumination and emotional valence of autobiographical memories (see Figs. 1 and 2). The role of positive and negative autobiographical memories as mediator variables was also tested using the bootstrap method.

The fit statistics of the model had a good fit in both age groups (*χ*^*2*^(4) = 2.40, *p* = 0.662; CFI = 1.00; TLI = 1.07; RMSEA = 0.00, and *χ*^*2*^(4) = 3.89, *p* = 0.421; CFI = 1.00; TLI = 1.01; RMSEA = 0.00, for young and older participants, respectively). The estimated parameters (presented as standardized) are shown in Fig. 1 for each age group. For the young participants, the results showed that depressive symptomatology was predicted by higher scores in rumination and fewer positive autobiographical memories. Additionally, negative memories were explained by higher scores in rumination and the paternal care and protection scales. In contrast, in the older group, in addition to rumination and positive memories, depressive symptoms were also predicted by higher scores in the maternal protection variable. Furthermore, negative memories were explained mainly by lower scores in rumination. None of the study variables reached statistical significance in explaining positive memories in either age group. Finally, no significant indirect effects were found of paternal/maternal care and protection variables on depressive symptoms through positive or negative autobiographical memories in either age group.

To ensure that the variables in the model are differently related as regards age group, a multi-group comparison was performed. Thus, the test of age equivalence involved the comparison of a model in which the factor loadings and covariations between the variables were allowed to vary across age groups (fully unconstrained) to a model that partially varied across groups (partially constrained) and to a fully constrained model.

The results of the model testing and model comparisons are presented in Table 2. As can be seen, the unconstrained model showed good fit statistics, suggesting that a common path model could be assumed across groups. We next proceeded to test whether further restrictions could be added to the model to make it more parsimonious and improve fit statistics. We performed chi-square difference tests for each path by comparing the unconstrained model with one in which a path was constrained to invariance across age groups. Thus, a series of models were compared in which each path was constrained to equality, one at a time, between groups. The change in chi-square between the fully unconstrained and the fully constrained models was significant.

**Table 2:** Comparative fit of tested models.

	Model fit indices						Model comparison tests		
Model	*df*	*X*^*2*^	*p*	RMSEA	CFI	AIC	*Δdf*	*ΔX*^*2*^	*p*(d)
1. Fully unconstrained	8	6.29	0.614	0.00	1.000	134.29			
2. Partially unconstrained	21	15.05	0.821	0.00	1.00	117.05			
Model 1 vs Model 2							13	8.76	0.791
3. Fully constrained	25	42.27	0.017	0.05	0.94	136.27			
Model 1 vs Model 3							17	35.98	0.005

A subsequent model was fitted in which all paths were constrained to invariance except the path found to differ significantly across groups: (1) the rumination to depressive symptoms path (*ΔX*^*2*^(1) = 4.08, *p* = 0.043); (2) the rumination to negative memories path (*ΔX*^*2*^(1) = 10.48, *p* = 0.001); (3) the paternal protection subscale to depressive symptoms path (*ΔX*^*2*^(1) = 4.14, *p* = 0.042); and (4) the paternal protection subscale to negative memories path (*ΔX*^*2*^(1) = 6.17, *p* = 0.013). This partially constrained model also provided a good fit. The change in chi-square between the unconstrained and partially constrained models was not significant (see Table 2), indicating no detectable differences in the model, in which all but four paths were constrained to equality across age groups. As the statistics of both models were similar, we computed the Akaike Information Criterion (AIC). The AIC allows the fit of different models to be compared and the model with the lowest AIC is selected as the one with the best fit. According to this criterion, the partially constrained model showed the best fit to the observed data.

According to the multi-group comparison, the relationship between rumination and depressive symptoms was significantly different for young and older participants. This relationship was stronger in the young group (rumination explained 16% of the variance in depressive symptomatology), compared to the older group (6.76% of variance explained). Regarding the rumination and negative memories path, in the young group, higher rumination scores increased negative memories (4.41% of variance explained), while in the older group, this relationship was reversed, that is, higher rumination decreased negative memories (3.24% of variance explained). The paternal protection dimension with depressive symptoms path, although significantly different between age groups, did not reach statistical significance in any model. Finally, regarding the paternal protection dimension with negative memories path, in the young group, higher scores in this dimension increased the number of negative memories (6.76% of variance explained) while, in the older group, this relation was not statistically significant (1% of variance explained).

## Discussion

The aim of the present study was to analyse whether the emotional valence of autobiographical memories (positive or negative) and parental bonds (care and control/protection) affect depressive symptomatology in a community sample, studying possible differences according to age group (young vs. old), as well as the influence of depressive rumination.

With regard to age, our results show that the younger adults, compared to the older adults, presented greater depressive symptomatology. This finding is consistent with previous studies reporting that, in the absence of clinical depression, older adults, experience fewer negatively valenced emotions than younger persons^93,94^. This is because, according to the Theory of Emotional Selectivity (TSS)^95^, as time horizons shorten with increasing age, motivational goals change, leading older people to focus on positive emotional experiences and to use greater resources and effective strategies for emotion regulation^96^. This might explain the steady decline in subclinical depression (or depressive symptomatology) from young to older adulthoood^97^. Similarly, young adults score higher on depressive rumination than older adults. Following previous studies such as that by Ricarte et al.^67^, older adults are less likely to engage in the use of negative cognitive strategies of emotion regulation, including rumination. However, in young people, rumination may not necessarily be a maladaptive strategy, since, at this stage of life, individuals might use it as a means of learning and reflecting on their emotional conflicts^67^.

In relation to autobiographical memories, the younger participants reported fewer positively valenced autobiographical memories and more negative memories compared to the older group. These findings are consistent with previous studies such as that by Ros and Latorre^51^ suggesting that older adults report fewer negative autobiographical memories than younger adults, despite being more likely to do so given their accumulation of negative experiences over time. This is due to older people being more motivated to remember their past in an emotionally satisfying way^95^. In fact, according to Wood and Conway^98^, older people generate a greater number of redemption sequences, i.e., past events that were originally negative come to be perceived as positive.

As for parental bonding, we have no knowledge of previous works exploring age differences in this regard. Our results show that the young adults scored higher on maternal care and lower on paternal protection, compared to their older counterparts, indicating more optimal parenting. This runs counter to the results of the meta-analysis by Gerlsma et al.^99^, which found individuals with poorer parental attachment present lower life satisfaction, as well as affective problems such as depression. However, in the present study, older adults have lower levels of depressive symptomatology despite having poorer parental attachment. Nonetheless, it should be borne in mind that the aforementioned study did not include samples aged over 65 years. In light of these results, it should be noted that, during the growth stages of the older adults in the present study, major cultural changes took place in Spain (e.g., Civil War, Franco's dictatorship). These might have had an impact on their evaluations of parental bonding, since the role of father and mother at that time may have differed from that of more recent times.

Focusing on the results obtained in the path analysis, these indicate that the greater the number of positive autobiographical memories, the lower is the probability of developing depressive symptomatology, i.e., positively valenced autobiographical memories appear to function as a protective factor for depressive symptoms. Moreover, this occurred in both young and older adults, coinciding with previous results^100,101^ in which the retrieval of happy autobiographical memories repaired sad mood in individuals who are not clinically depressed.

With respect to depressive rumination, this significantly increases depressive symptomatology in older adults and, above all, in young adults. The Response Styles Theory (RST)^59^ holds that depressive rumination is a tendency similar to a stable, enduring and habitual trait that allows a person to engage in repetitive self-focus in response to a depressed mood. In this way, depressive rumination enhances mood-congruent negative thinking and hinders the development of instrumental problem-solving behaviour^62^, as well as hampering engagement in pleasurable activities that could help a person escape from their dysphoric mood, thus prolonging the duration, and increasing the severity of, depressive symptoms^102^.

Furthermore, with regard to parental ties, we observed that overprotection by mothers is associated with greater depressive symptomatology in older adults. The behaviour of such mothers is characterized by being overprotective, that is, not allowing children to take their own decisions. In the same line, previous studies have underlined the fact that high levels of overprotection by the mother, but not by the father, are linked to mood disorders such as depression^19^. This may be due to the quality of maternal bonding as a predictor of depressive symptomatology being more consistent than paternal bonding, supported by the fact that maternal bonding plays a more important role in individuals’ psychological development, especially in early childhood^21^. It should also be noted that, during the childhood of today’s older adults, traditional gender roles were followed, with mothers assuming the role of primary caregivers, spending more time with their children in the absence of their fathers. Thus, the perception of the bonds established with parents (during childhood and part of adolescence) only has a direct effect on depressive symptomatology in the case of older people. These results should be considered when implementing interventions designed to prevent or minimize depressive symptoms in this population group.

Despite the literature suggesting that parental bonding may increase people's vulnerability to emotional dysfunction through dysfunctional cognition (based on negative information processing structures)^103^, in our study, the valence of autobiographical memories does not affect the relationship between parental bonding and depressive symptoms. These findings may, however, be due to our participants generally presenting low depressive symptomatology, which, furthermore, was measured on the basis of symptoms experienced in the last two weeks. Therefore, it is necessary to replicate this study with samples with greater variation in terms of depressive symptomatology.

Regarding the relationship between parental bonds and autobiographical memories, we find that, in the group of young adults, higher scores on paternal care and protection predict a greater number of negative autobiographical memories. In this sense, it has been suggested that perceptions of fathers tend to be more personal than those of mothers, especially as regards memories of negative situations^104^. Thus, childhood memories associated with fathers are more prominent, particularly when recalling negative situations. Moreover, memories of fathers associated with negative situations have a more adverse effect as the negative impression of fathers is more permanently embedded^104^.

Although parental bonding is something we cannot modify, since it forms part of a person's past history, autobiographical memories can be modified by accentuating positive or negative details (e.g., in terms of emotional intensity)^105^. Thus, autobiographical memories allow for the construction of redemptive life narratives, where negative past events (associated with bad experiences, obstacles, failures, losses, etc.) are redeemed by positive outcomes, generating positive meanings and feelings of gratitude, growth, etc.^106^.

Our results show that, depending on the age group, the influence of the parental bond centres on either the mother or the father figure. In this sense, in the study by Yaffe^107^, it is suggested that children’ perceptions of the parenting styles of fathers and mothers differ, with mothers being more positively perceived than fathers in relation to emotional aspects, while fathers tend to be perceived as more authoritarian, rigid, severe, restrictive and formal. However, the lack of conclusive results justifies the need for further studies in this regard. In addition, it is worth noting the differences in parenting styles received by the older group compared to the younger group. In this sense, among our older participants, mothers were largely the main caregivers, spending more time with the children, while fathers worked outside the home. Thus, they might reasonably have a larger number of memories associated with their female parents. Notwithstanding all of the above, dysfunctional parental styles clearly have an impact across the life course^108^, especially on mental health.

We also found a relationship between depressive rumination and increased negative autobiographical memories in our young adults, while in the older adults this relationship was inverse, i.e., the greater the depressive rumination, the lower was the number of negative autobiographical memories. The results found in the young adult group support previous findings reporting an association between rumination and greater recall of negative autobiographical information^109^. A possible explanation for the differences between groups is that individuals who engage in depressive rumination tend to focus on themselves in response to negative mood states (their depressive symptoms and their consequences), resulting in their being more prone to retrieve and re-experience distressing past events^110^. That is, individuals who engage in self-centred ruminative thinking are more likely to generate negative events when asked to freely recall autobiographical memories^111^. In this sense, as mentioned, young people have a greater tendency to ruminate on their own emotional conflicts, acting as a means of learning, which might lead to greater access to negative autobiographical memories. This is not the case for older adults, as they are more prone than their younger counterparts to concerns that are less directly related to themselves and, therefore, are not so associated with the memory of past autobiographical events, but rather with the health and well-being of their loved ones^112^. Nonetheless, our results in the group of older adults run counter to those obtained by most previous studies that suggest a link between rumination and the retrieval of negative autobiographical memories^111^. However, it is worth noting that most of these studies focus on clinical samples, whereas the present study uses a community sample of individuals with no diagnosis of depression.

The present study is not without limitations, with one being the possible lack of representativeness in the sample of young adults as they are all university students. Future studies should take this limitation into account. In addition, such studies should also include the analysis of possible gender-related differences, as men and women may perceive the role of their caregivers differently^113^. Moreover, in our study, the content of the autobiographical memories generated by the participants was not taken into account, and so it would be of interest for future studies to consider this, as well as the time of life related to these memories. It would also be useful for future studies to collect information about trauma or adverse experiences during childhood, from which to gain greater insight into the age-related differences found.

## Conclusions

Depression is a disease with serious consequences for persons who suffer from the disorder, such as feelings of helplessness, despair, stigma, worthlessness, fear, vulnerability and, consequently, low quality of life. Hence, it is important to continue advancing in the analysis and understanding of all factors that may influence its onset and maintenance of depressive symptomatology.

Our results show that the role of parents, and in particular poor parental attachment, may lead an individual to be more vulnerable to depressive symptomatology. This suggests the need for further research on effective preventive interventions to promote parenting styles based on care and protection of children (for more information on such interventions see, Wright & Edginton^114^).

Furthermore, as regards emotion regulation, we identified two strategies that yield different results. Depressive rumination is a risk factor in the development of depressive symptoms, while access to positive autobiographical memories is a protective factor. Therefore, both factors should be considered when establishing interventions aimed at preventing and improving depressive symptoms, especially the promotion of procedures that facilitate access to this type of memory.

## Data Availability

Access data can be obtained upon reasonable request by contacting the corresponding author of this paper (Dolores Fernández-Pérez).

## References

[1] American Psychiatric Association. *Diagnostic and Statistical Manual of Mental Disorders*. 5th ed. Arlington, VA: American Psychiatric Association (2013).

[2] World Health Organization. (2021). *Depression*. https://www.who.int/me hat affects almost 300 million people, causing an enormous burden worldwidediacentre/factsheets/fs369/en/.

[3] Ayuso-Mateos JL, Nuevo R, Verdes E, Naidoo N, Chatterjo S (2010). From depressive symptoms to depressive disorders: The relevance of thresholds. Br. J. Psychiatry..

[4] Lyvers M, Mayer K, Needham K, Thorberg FA (2019). Parental bonding, adult attachment, and theory of mind: A developmental model of alexithymia and alcohol-related risk. J. Clin. Psychol..

[5] Cassidy, J. The nature of the child’s ties. In J. Cassidy & P.R. Shaver (Eds.). *Handbook of Attachment: Theory, Research, and Clinical Applications* (pp. 3–22). New York: Guilford Press (2008).

[6] Bowlby J (1969). Attachment and Loss: Attachment.

[7] Suzuki H, Kitamura T (2011). The Parental Bonding Instrument: A four-factor structure model in a japanese college simple. Open Fam. Stud. J..

[8] Ohtaki Y (2017). Parental bonding during childhood affects stress-coping ability and stress reaction. J. Health Psychol..

[9] Bartholomew K, Horowitz LM (1991). Attachment styles among young adults: A test of a four-category model. J. Pers. Soc. Psychol..

[10] Parker G, Tupling H, Brown LB (1979). A parental bonding instrument. Br. J. Med. Psychol..

[11] Parker G (1983). Parental ‘affectionless control’ as an antecedent to adult depression: A risk factor delineated. Arch. Gen. Psychiatry.

[12] Parker G (1984). The measurement of pathogenic parental style and its relevance to psychiatric disorder. Soc. Psychiatry.

[13] Gladstone, G. L., & Parker, G. B. The role of parenting in the development of psychopathology: An overview of research using the Parental Bonding Instrument. In Hudson, J. L. & Rapee, R. M. (Eds.), *Psychopathology and the family* (pp. 21–33). New York, NY: Elsevier Science (2005).

[14] Murphy E, Wickramaratne P, Weissman M (2010). The stability of parental bonding reports: A 20-year follow-up. J. Affect Disord..

[15] Wel F, Linssen H, Abma R (2000). The parental bond and the well-being of adolescents and young adults. J. Youth Adolesc..

[16] Bowlby J (1988). A secure base: Clinical applications of attachment theory.

[17] Mikulincer M, Shaver PR (2012). An attachment perspective on psychopathology. World Psychiatry.

[18] Bowlby J, Ainsworth M, Retherton I (1992). The origins of attachment theory. Dev. Psychol..

[19] Enns MW, Cox BJ, Clara I (2002). Parental bonding and adult psychopathology: results from the US national comorbidity survey. Psychol. Med..

[20] Tugnoli, S., Casetta, I., Caraccciolo, S., & Salviato, J. Parental bonding, depression, and suicidal ideation in medical students. *Front. Psychol*. **4,** 13:877306; 10.3389/fpsyg.2022.877306 (2022). 10.3389/fpsyg.2022.877306 PMC9386274 35992478

[21] Yen J, Tam CL, Lee SL (2021). Parental bonding, depressive experiences, and symptomatology: An investigation among college students in Malaysia. Psych. J..

[22] Abbaspour A, Bahreini M, Akaberian S, Mirzaei K (2021). Parental bonding styles in schizophrenia, depressive and bipolar patients: A comparative study. BMC Psychiatry.

[23] Morford A (2017). Major depression, childhood trauma, parenting styles and oxidative stress: A well-controlled study in unmedicated individuals. Biol. Psychiatry.

[24] Johnstone JM (2016). Childhood predictors of lifetime suicide attempts and non-suicidal self-injury in depressed adults. Aus. N. Z. J. Psychiatry.

[25] Dinger U (2015). Interpersonal problems, dependency, and self-criticism in major depressive disorder. J. Clin. Psychol..

[26] Ono Y, Takaesu Y, Nakai Y, Ichiki M, Masuya J, Kusumi I, Inoue T (2017). The influence of parental care and overprotection, neuroticism and adult stressful life events on depressive symptoms in the general adult population. J. Affect. Disord..

[27] Shaw BA, Krause N, Chatters L, Connell C, Ingersoll-Dayton B (2004). Emotional support from parents early in life, aging, and health. Psychol. Aging.

[28] Rossi, A. S., & Rossi, P. H. *Of human bonding: Parent– child relations across the life course*. New York, NY: de Gruyter (1990).

[29] Turner HA, Muller PA (2004). Long-term effects of child corporal punishment on depressive symptoms in young adults. J. Fam. Issues.

[30] Köber C, Facompré CR, Waters TEA, Simpson JA (2019). Autobiographical memory stability in the context of the adult attachment interview. Cognition.

[31] Fivush, R., & Graci, M. E. Autobiographical memory In Byrne, J. H. (Series Ed.), *Learning and Memory: A Comprehensive Reference: Vol. 1. Learning Theory and Behavior* (Wixted, J., Editor for Cognitive Psychology of Memory section), 2e, Elsevier, Oxford (2017).

[32] Nelson K, Fivush R (2004). The emergence of autobiographical memory: A social cultural developmental theory. Psychol. Rev..

[33] Fivush, R., Haden, C., & Reese, E. Remembering recounting, and reminiscing: The development of autobiographical memory in social context. In Rubin, D. R. (Ed.), *Remembering Our Past: Studies in Autobiographical Memory* (pp. 341–359). New York: Cambridge University Press (1996).

[34] Wolf T, Zimprich D (2016). The distribution and the functions of autobiographical memories: Why do older adults remember autobiographical memories from their youth?. Eur. J. Ageing.

[35] Pillemer DB (2003). Directive functions of autobiographical memory: The guiding power of the specific episode. Memory.

[36] Alea N, Bluck S (2003). Why are you telling me that? A conceptual model of the social function of autobiographical memory. Memory.

[37] Bluck S, Li KZH (2001). Predicting memory completeness and accuracy: Emotion and exposure in repeated autobiographical recall. Appl. Cogn. Psychol..

[38] Ford JH, Addis DR, Giovanello KS (2012). Differential effects of arousal in positive and negative autobiographical memories. Memory.

[39] Hitchcock C, Newby J, Timm E, Howard RM, Golden A, Kuyken W, Dalgleish T (2020). Memory category fluency, memory specificity, and the fading affect bias for positive and negative autobiographical events: Performance on a good day-bad day task in healthy and depressed individuals. J. Exp. Psychol. Gen..

[40] Rassmussen AS (2009). Emotional valence and the functions of autobiographical memories: Positive and negative memories serve different functions. Mem. Cognit..

[41] Dalgleish T, Hitchcock C (2023). Transdiagnostic distortions in autobiographical memory recollection. Nat. Rev. Psychol..

[42] Crane C, Barnhofer T, Visser C, Nightingale H, Williams JMG (2007). The effects of analytical and experiential rumination on autobiographical memory specificity in individuals with a history of major depression. Behav. Res. Ther..

[43] Lewis PA, Critchley HD, Smith AP, Dolan RJ (2005). Brain mechanisms for mood congruent memory facilitation. Neuroimage.

[44] Hamilton JP, Gotlib IH (2008). Neural substrates of increased memory sensitivity for negative stimuli in major depression. Biol. Psychiatry.

[45] Luchetti M, Sutin AR (2018). Age differences in autobiographical memory across the adult lifespan: Older adults report stronger phenomenology. Memory.

[46] Ros L, Latorre JM, Serrano JP, Ricarte JJ (2017). Overgeneral autobiographical memory in healthy young and older adults: Differential age effects on components of the capture and rumination, functional avoidance, and impaired executive control (CaRFAX) model. Psychol. Aging.

[47] Wilson FCL, Gregory JD (2018). Overgeneral autobiographical memory and depression in older adults: A systematic review. Aging Ment. Health..

[48] Devitt AL, Addis DR, Schacter DL (2017). Episodic and semantic content of memory and imagination: A multilevel analysis. Mem. Cognit..

[49] Ford JH, DiGirolamo MA, Kensinger EA (2016). Age influences the relation between subjective valence ratings and emotional word use during autobiographical memory retrieval. Memory.

[50] Berntsen D (2002). Tunnel memories for autobiographical events: Central details are remembered more frequently from shocking than from happy experiences. Mem. Cognit..

[51] Ros L, Latorre JM (2010). Gender and age differences in the recall of affective autobiographical memories using the autobiographical memory test. Pers. Individ. Diff..

[52] Carstensen LL, Pasupathi M, Mayr U, Nesselroade J (2000). Emotional experience in everyday life across the adult life span. J. Pers. Soc. Psychol.

[53] Mather M, Carstensen LL (2005). Aging and motivated cognition: The positivity effect in attention and memory. Trends Cogn. Sci..

[54] Grühn D, Smith J, Baltes PB (2005). No aging bias favoring memory for positive material: Evidence from a heterogeneity-homogeneity list paradigm using emotionally toned words. Psychol. Aging.

[55] Rubin DC (2006). The Basic-Systems Model of Episodic Memory. Perspect. Psychol. Sci..

[56] Kennedy Q, Mather M, Carstensen LL (2004). The role of motivation in the age-related positivity effect in autobiographical memory. Psychol. Sci..

[57] Joormann J, Stanton CH (2016). Examining emotion regulation in depression: A review and future directions. Behav. Res. Ther..

[58] Emery L, Sorrell A, Miles C (2020). Age differences in negative, but not positive rumination. J. Gerontol. B.

[59] Nolen-Hoeksema S (1991). Responses to depression and their effects on the duration of depressive episodes. J. Abnorm. Psychol..

[60] Watkins ER (2008). Constructive and unconstructive repetitive thought. Psychol. Bull..

[61] Holmes EA, Mathews A (2010). Mental imagery in emotion and emotional disorders. Clin. Psychol. Rev..

[62] Nolen-Hoeksema S, Wisco BE, Lyubomirsky S (2008). Rethinking rumination. Perspec Psychol. Sci..

[63] Slofstra C, Eisma MC, Holmes EA, Bockting CLH, Nauta MH (2017). Rethinking a negative event: the affective impact of ruminative versus imagery-based processing of aversive autobiographical memories. Front. Psychol..

[64] Williams JMG (2007). Autobiographical memory specificity and emotional disorder. Psychol. Bull..

[65] Sumner J (2010). Overgeneral autobiographical memory as a predictor of the course of depression: A meta-analysis. Behav. Res. Ther..

[66] Thomsen DK, Schnieber A, Olesen MH (2011). Rumination is associated with the phenomenal characteristics of autobiographical memories and future scenarios. Memory.

[67] Ricarte JJ, Ros L, Serrano JP, Martínez-Lorca M, Latorre JM (2016). Age differences in rumination and autobiographical retrieval. Aging Ment. Health..

[68] Mallers MH, Charles ST, Neupert SD, Almeida DM (2010). Perceptions of childhood relationships with mother and father: Daily emotional and stressor experiences in adulthood. Dev. Psychol..

[69] Bauer PJ, Tasdemir-Ozdes A, Larkina M (2014). Adults’ reports of their earliest memories: Consistency in events, ages, and narrative characteristics over time. Conscious Cogn..

[70] Hinnen C, Sanderman R, Sprangers MAG (2009). Adult attachment as mediator between recollections of childhood and satisfaction with life. Clin. Psychol. Psychother..

[71] Cunha M, Martinho MI, Xavier AM, Espírito Santo H (2013). Early memories of positive emotions and its relationships to attachment styles, self-compassion and psychopathology in adolescence. Eur. Psychiatry.

[72] Brown J, Pengas G, Dawson K, Brown LA, Clatworthy P (2009). Self-administered cognitive screening test (TYM) for detection of Alzheimer´s disease cross sectional study. BMJ.

[73] Ferrero-Arias J, Turrión-Rojo MA (2016). Validation of a Spanish version of the test your memory. Neurol..

[74] Cella D (2010). The patient-reported outcomes measurement information system (PROMIS) developed and tested its first wave of adult self-reported health outcome item banks. J. Clin. Epidemiol..

[75] Brown J, Pengas G, Dawson K, Brown LA, Clatworthy P (2009). Self-administered cognitive screening test (TYM) for detection of Alzheimers disease cross sectional study. BMJ Br. Med. J..

[76] Pilkonis PA (2011). Item banks for measuring emotional distress from the patient reported outcomes measurement information system (PROMIS): Depression, anxiety and anger. Assessment.

[77] Wilhelm K, Niven H, Parker G, Hadzi-Pavlovic D (2005). The stability of the parental bonding instrument over a 20-year period. Psychol. Med..

[78] Williams JMG, Barnhofer T, Crane C, Beck AT (2005). Problem solving deteriorates following mood challenge in formerly depressed patients with a history of suicidal ideation. J. Abnorm. Psychol..

[79] Williams JMG, Broadbent K (1986). Autobiographical memory in suicide attempters. J. Abnorm. Psychol..

[80] Beck AT, Ward CH, Mendelson M, Mock J, Ernaugh J (1961). An inventory for measuring depression. Arch. Gen. Psychiatry.

[81] Sanz, J., & Vazquez, C. *BDI-II, Inventario de Depresión de Beck-II*. Pearson (2011).

[82] Kendall CP, Hollon SD, Beck A, Hammen C, Ingram RE (1987). Issues and recommendations regarding use of the beck depression inventory. Cognit. Ther. Res..

[83] Raes F, Hermans D, Williams JMG, Eelen P (2007). A sentence completion procedure as an alternative to the autobiographical memory test for assessing overgeneral memory in non-clinical populations. Memory.

[84] Ricarte JJ, Aizpurúa E, Ros L, Latorre JM, Raes F (2018). Psychometric properties of the spanish short depressive rumination scale in a nonclinical simple. Span J. Psychol..

[85] Raes F, Hermans D, Williams JMG, Bijttebier P, Eelen P (2008). A “Triple W”- model of rumination on sadness: Why am I feeling sad, what’s the meaning of my sadness, and wish I could stop thinking about my sadness (but I can’t!). Cognit. Ther. Res..

[86] Conway M, Csank PAR, Holm SL, Blake CK (2000). On assessing individual differences in rumination on sadness. J. Pers. Assess..

[87] Nolen-Hoeksema S, Morrow J (1991). A prospective study of depression and posttraumatic stress symptoms after a natural disaster: The 1989 Loma Prieta earthquake. J. Pers. Soc. Psychol..

[88] Byrne BM (2001). Structural equation modeling with AMOS: Basic concepts, applications and programming.

[89] Bentler PM (1992). On the fit of models to covariances and methodology to the bulletin. Psychol. Bull..

[90] Preacher KJ, Hayes AF (2008). Asymptotic and resampling strategies for assessing and comparing indirect effects in multiple mediator models. Behav. Res. Met..

[91] Avagianou P, Zafiropoulou M (2008). Parental bonding and depression: Personality as a mediating factor. Int. J. Adolesc. Med. Health.

[92] Yu T, Hu J (2022). Parental bonding and depressive symptoms among Chinese college students during the COVID-19 pandemic: The roles of neuroticism and social support. Open. J. Soc. Sci..

[93] Charles, S. T., & Carstensen, L. L. Emotion regulation and aging In Gross, J. J. (Ed.), *Handbook of Emotion Regulation* (pp. 307–327). (The Guildford Press, 2007).

[94] Consedine, N. S., & Magai, C. Emotional development in adulthood: A developmental functionalist review and critique In Hoare, C. (Ed.), *Handbook of Adult Development And learning* (pp. 123–148). Oxford University Press (2006).

[95] Carstensen, L. L. Motivation for social contact across the life span: A theory of socioemotional selectivity In Jacobs, J. E. (Ed.), *Developmental Perspectives on Motivation* (pp. 209–254). Lincoln, NE: University of Nebraska Press (1993). 1340521

[96] Carstensen LL, Fung HH, Charles ST (2003). Socioemotional selectivity theory and the regulation of emotion in the second half of life. Motiv. Emot..

[97] Wuthrich VM, Johnco CJ, Wetherell JL (2015). Differences in anxiety and depression symptoms: Comparison between older and younger clinical samples. Int. Psychogeriatr..

[98] Wood WJ, Conway M (2006). Subjective impact, meaning making, and current and recalled emotions for self-defining memories. J. Pers..

[99] Gerlsma C, Emmelkamp PMG, Arrindell WA (1990). Anxiety, depression, and perception of early parenting: A meta-analysis. Clin. Psychol. Rev..

[100] Joorman J, Siemer M, Gotlib IH (2007). Mood regulation in depression: Differential effects of distraction and recall of happy memories on sad mood. J. Abnorm. Psychol..

[101] Latorre JM (2015). Life review based on remembering specific positive events in active aging. J. Aging Health.

[102] Huang L (2022). Higher rumination tendency is associated with reduced positive effects of daily activity participation in people with depressive disorder. Occup. Ther. Int..

[103] Williams CL (2015). Specificity of parental bonding and rumination in depressive and anxious emotional distress. Pers. Ind. Diff..

[104] Kim ES, Kim HE, Kim J (2020). The neural influence of autobiographical memory related to the parent-child relationship on psychological health in adulthood. PLoS ONE.

[105] Sutin A, Gillath O (2009). Autobiographical memory phenomenology and content mediate attachment style and psychological distress. J. Couns. Psychol..

[106] McAdams DP, Logan RL, Reischer HN (2022). Beyond the redemptive self: Narratives of acceptance in later life (and in other contexts). J. Res. Pers..

[107] Yaffe, Y. Systematic review of the differences between mothers and fathers in parenting styles and practices. *Curr. Psychol*. s1214 4-020-01014-6 (2020).

[108] Burns RA, Loh V, Byles JE, Kending HL (2018). The impact of childhood parental quality on mental health outcomes in older adults. Aging Ment. Health.

[109] Connolly SL, Alloy LB (2018). Negative event recall as a vulnerability for depression: Relationship between momentary stress-reactive rumination and memory for daily life stress. Clin. Psychol. Sci..

[110] Cappeliez P, O’Rourke N, Chaudhury H (2005). Functions of reminiscence and mental health in later life. Aging Ment. Health..

[111] Lyubomirsky S, Caldwell ND, Nolen-Hoeksema S (1998). Effects of ruminative and distracting responses to depressed mood on retrieval of autobiographical memories. J. Pers. Soc. Psychol..

[112] Gonçalves DC, Byrne GJ (2013). Who worries most? Worry prevalence and patterns across the lifespan. Int. J. Geriatr. Psychiatry.

[113] Spence JT (1993). Gender-related traits and gender ideology: Evidence for a multifactorial theory. J. Pers. Soc. Psychol..

[114] Wright B, Edginton E (2016). Evidence-based parenting interventions to promote secure attachment: Findings from a systematic review and meta-analysis. Glob. Pediatr. Health..

